# Vestibular evoked myogenic potential: recording methods in humans and guinea pigs

**DOI:** 10.1016/S1808-8694(15)31389-6

**Published:** 2015-10-17

**Authors:** Aline Cabral de Oliveira, Ricardo David, José Fernando Colafêmina

**Affiliations:** 1 Speech and hearing therapist. Graduate student from the Medical School of Ribeirão Preto - USP; 2 MS. in Physicis applied to Medicine - USP - Ribeirão Preto. Graduate student - Medical School of Ribeirão Preto - USP; 3 PhD. Associate Professor - USP - Ribeirão Preto. Assistant Professor - Medical School of Ribeirão Preto - USP. Faculdade de Medicina de Ribeirão Preto - USP

**Keywords:** electromyography, methods, evoked potentials auditory

## Abstract

The vestibular evoked myogenic potential (VEMP) is a clinical test that assess the vestibular function by means of an inhibitory vestibulo-neck reflex, recorded in body muscles in response to high intensity acoustic stimuli.

**Aim:**

To check and analyze the different methods used to record VEMPs in humans and in guinea pigs.

**Materials and Methods:**

We researched the following databases: MEDLINE, LILACS, SCIELO and COCHRANE.

**Results:**

we noticed discrepancies in relation to the ways used to record the vestibular evoked myogenic potentials in relation to the following factors: patient position at the time of recording, type of sound stimulus used (clicks or tone bursts), parameters for stimuli mediation (intensity, frequency, duration of presentation, filters, response amplification gain and windows for stimulus recording), type of phone used and way of stimulus presentation (mono or binaural, ipsi or contralateral).

**Conclusion:**

There is no consensus in the literature as to the best recording method for vestibular evoked myogenic potentials. We need more specific studies in order to compare these recordings and establish a standard model to use it in the clinical practice.

## INTRODUCTION

The Vestibular Evoked Myogenic Potential (VEMP), is a clinical test that assesses the vestibular function through an inhibitory vestibular-neck reflex that is recorded from body muscles in response to the acoustic stimulation of the saccule^1^, ^2^, ^3^, ^4^.

Historically, vestibular evoked potentials by sound stimulation were researched by investigators of the cortical auditory evoked potentials^5^, ^6^, ^7^. Initially, to record these potentials, the active electrodes were positioned on the scalp: inion and retroauricular region. Reference electrodes were positioned on the ear lobe or on the nose. Positioning the active electrode on the inion allowed us to record the electromyographic activity of the occipital muscles, and when placed on the retroauricular region, it recorded the activity of the posterior retroauricular muscle. According to these authors, retroauricular responses were less uniform than those from the inion and were present in just a few individuals with normal hearing. This finding has been attributed to the lingering characteristic of the retroauricular muscle, with great interpersonal variation^7^. These authors also observed that recording these potentials in the trapezius muscle, which is a posterior neck muscle, was similar to recording on the inion. In recording the vestibular evoked potential, the placement of the active electrode on the studied muscle group increases the muscle potentials in lieu of the neural potentials that happen simultaneously^5^.

Back in 1992, a study^1^ was carried out with VEMP recording on the sternocleidomastoid muscles, interpreting them similarly to the recording on the posterior neck muscles. In 1997, Other authors^8^ recorded this potential on the anterior neck muscles (sternocleidomastoid muscle) and posterior (trapezius muscle) in normal hearing individuals. In this study we noticed differences associated with the absolute amplitude and latencies of curve peaks; however, they did not report on the intrinsic advantages and disadvantages of each method.

Despite the numerous studies in the filed of vestibulo-evoked myogenic potentials (VEMP), many recording techniques have been developed and such tests can be recorded through many types of stimuli and from different body muscles.

Having this brief introduction, the goal of the present investigation was, through a bibliographic survey, to check and analyze the different methods available to record vestibular evoked myogenic potentials in men and in lab animals.

## MATERIALS AND METHODS

This study was carried out through an electronic search in the data bases: MEDLINE, LILACS, SCIELO and COCHRANE library, on papers published between 1964 and 2007, based on the keyword: VEMP.

We carried out a broad electronic search encompassing only papers which described the detailed method do record VEMPs. The techniques hereby reported were empirically experimented in an otolaryngology ward in the city of Ribeirão Preto - SP and will be described in a later paper.

## RESULTS

We found 105 papers in our electronic search between 1964 and 2007. Among these, only 47 were within the pre-established criteria for the sample.

Based on the research mentioned, we found discrepancies as to the means used to record vestibular evoked myogenic potentials. The most important parameters to be considered when recording VEMPs are:

### Test equipment

Any device capable of recording middle latency evoked potentials, when properly configured, can be used to record VEMPs. In our experiments, we used the following models: Nicolete CA 2000 (CITO HM 8510 IMPR.), ATI Nautilus PE version 4.19 LERMEC S.R.L Bio-Logic System Corp. Mod Traveller E-UNIT, besides an experimental prototype, which will be described in a later paper.

Equipment with only one recording channel are able to record such potentials. However, using two or more channels can provide extra resources, such as the possibility to compare records between two sides^9^, ^10^, ^11^. This joint analysis of the two brain hemispheres can be carried out through the possibility of averaging out monoaural and binaural stimuli, through the use of earphones^9^, ^12^ or through bone vibrators (placed on the mastoid, directly behind the pinna)^13^.

The device has to be adjusted to record middle latency potentials, with a 100ms window (10ms per division), which is standard in most studies^13^, ^14^, ^15^. However, in some cases, we saw recordings in the following windows: 50, 60 and 80ms^10^, ^16^, ^17^, ^18^, ^19^.

### Stimuli averaging

Sound stimuli may be presented through ear phones or intra-canal phones^16^, ^21^, ^20^, when the stimuli passes through air conduction or through a bone vibrator (placed on the mastoid process), when the stimulus is provided through the bone^13^, ^15^, ^22^, ^23^. Usually, the recordings are carried out using surface electrodes^13^, ^15^, ^16^, ^18^, ^24^, of the circular type, with a diameter above 8mm^20^.

The stimuli may be averaged by means of tone bursts (in the frequencies of 250Hz25, 500Hz^13^, ^15^, ^19^, ^25^, ^26^, ^27^, ^28^, 1000Hz^25^, ^28^ 3000Hz^10^, 5000Hz^15^, ^21^) or clicks^2^, ^9^, ^11^, ^14^, ^16^, ^17^, ^19^, ^21^, ^22^, ^24^, ^29^, which shall be employed at intensities above 75 dBHL. Currently, stimuli above 90 dBHL are used^2^, ^8^, ^9^, ^10^, ^11^, ^14^, ^15^, ^16^, ^18^, ^19^, ^24^, ^28^, ^29^, ^30^, ^31^. However, in many studies, the threshold is surveyed by means of stimuli of different intensities (with ascending or descending techniques) until the lowest stimulus intensity capable of triggering a response is found^2^, ^21^, ^25^, ^32^.

The recording is done by means of averaging or adding, and the parameters used in most studies are equal to or higher than 200 stimuli^2^, ^8^, ^9^, ^10^, ^14^, ^17^, ^20^, ^26^, ^27^, ^30^, ^31^. In general, the higher the number of stimuli and the lower the shooting rate (number of stimuli per second), the better the recording quality. Nonetheless, these alterations tend to increase the recording time.

In order to curb artifacts and interferences in signal recording, we used band pass filters going from 20 to 2000Hz^15^, ^16^, ^17^, ^18^, ^19^, ^22^, ^24^, ^27^, ^28^. However, in the literature we also found band pass filters going from 10 to 2500Hz^10^; 10 to 3000Hz^21^, 10 to 2000Hz^25^, ^31^, 20 to 2500Hz^13^, 15 to 20000Hz^26^ and 30 to 3000Hz^20^.

It was only in one study^27^, that we had a contralateral masking of the stimulus, using a band pass noise.

### Test room and patient preparation

VEMPs must be recorded in a silent room (there is no need to be sound proof)^8^, ^21^, ^10^, under mild and uniform temperature, and the patient must be sitting in a comfortable chair or laying down on a bed. The patient and/or family members must be educated about the procedures carried out during the test in order to avoid possible physical or emotional stress, which can interfere in the recordings.

The patient’s skin must be cleaned with an alcohol soaked cotton pad. Afterwards, the electrodes must be affixed with adhesive tape, after prior use of electrolytic paste10.

### Test procedures

#### Recording at the vertex and inion

In these types of recordings, the reference lead is affixed to the ear lobe, the live is placed on the vertex (highest point of the head) or on the inion (most evident medial surface of the occipital bone base). The reference leads are placed on the surface of the spinal process of the seventh neck vertebra and the ground lead is placed on the forehead^27^. The central positioning of the leads in relation to the skull places them in an equidistant position in relation to the right and left vestibular systems, preventing the concurrent differentiation between both hemispheres.

For proper recording, it is necessary that the patient push his/her head back, using the back of the neck^5^, ^6^, ^33^. In order to do this, we use a cuff placed between the back of the neck and the chair’s head rest and we set the pressure through the device’s pressure gauge, and in our studies we used the value of 20mm of mercury as standard pressure. However, the use of such device to control muscle tone must not be used often, because it suffers interferences which are intrinsic to the method, such as: variable cuff placement in relation to the head, lack of cuff pressure initial control and the way the cuff is placed next to the individual’s body.

The recording of potentials in these anatomical regions do not have proven clinical applications, because there are factors that impact such recording. Among them we have: positioning the leads in the medial portion of the skull, equidistant between the left and right vestibular systems (very close to each other), which impairs the comparison between the two hemispheres, especially with the binaural stimulus. Another issue is the placement of the leads on the head, which is impaired by the presence of hair, requiring special care or localized hair removal, which would surely bother patients.

### Recording on the trapezius muscle

In order to record VEMPs on the trapezius muscle, the live leads must be positioned at the level of the 5th and 6th neck vertebrae (at approximately 1.5cm from the vertical line center of the vertebral spine), the reference leads on the medial portion of the clavicle line and the ground lead on the center of the forehead^2^, ^34^.

During the recordings of this myogenic potential, the patient must remain seated with the cuff placed on the head rest of his/her chair, which will serve to check the patient’s strength during the test. This strength is intrinsically associated to an increase in trapezius muscle contraction, which enhances the recording of such potential. Thus, we pump a small amount of air in the cuff and them the patient pushes his/her head against it (towards the chair’s head rest) until the pressure gauge reads 20mm of mercury, which must be controlled by the patient in order to keep this value constant.

Another method employed to record VEMPs in the trapezius muscle would be to position the patient laying belly down, with the head outside the bed’s limits in such a way that the patient must keep the head up, thus naturally contracting the trapezius^2^, ^34^.

### Recording on the sternocleidomastoid muscle

In order to record VEMPs in this muscle, the active lead must be placed on the upper half of the sternocleidomastoid muscle, ipsilateral to the stimuli^2^, ^8^, ^13^, ^14^, ^17^, ^19^, ^20^, ^25^, ^27^, ^28^. As to the placement of the reference lead, the following positions have been reported: on the upper border of the sternum^2^, ^13^, ^14^, ^17^, ^19^, ^20^, ^24^, ^27^, ^28^, ^32^, on the sternocleidomastoid muscle tendon^25^ or on the upper clavicle border^26^. In most studies, the ground electrode must be positioned on the forehead middle line^10^, ^13^, ^14^, ^15^, ^16^, ^18^, ^24^, ^25^, ^26^, ^27^, ^31^.

The patient must remain seated, with maximum head lateral rotation to the contralateral side of the stimulus, in order to active the muscle, and with the eyes line parallel to the floor^9^, ^10^, ^13^, ^16^, ^17^, ^18^, ^19^, ^24^, ^25^, ^27^. It is common for the patient to naturally rest the chin on the shoulder, and this is not correct.

Some authors^30^, ^26^ report another technique for the simultaneous bilateral recording (on the ipsi and contralateral muscles), in which the patient is laying down on his/ her belly with the head up without a rest, thus allowing proper muscle tension for the recording^17^, ^26^, ^35^. Another recording possibility is to ask the patient to, in this position, raise the head and flex the back of the neck in order to obtain a bilateral contraction of the sternocleidomastoid muscles^26^.

### Recording on the head’s splenium muscle

In order to record VEMPs from this muscle, the patient must be laying belly down and must keep the head up, looking forward. The live leads must be placed, approximately, on the middle line between the mastoid process and the spinal process of the seventh neck vertebra (C7), where the muscle may be palpated against the resistance. And the reference lead on the C7 spinal process^20^.

### Recording from the upper and lower limbs

In the literature we see this recording in upper and lower limbs, and a high number of stimuli are necessary (usually above one thousand), with four or five sessions, which will result in one single biphasic wave, with middle latency between 40 and 60ms. Muscle contraction power and intensity (during recording) are directly related to the amplitude of the recorded wave^5^.

### Recording from the retroauricular muscle

Recording from this muscle is rarely recommended because of its lingering nature, and this makes it nonfunctional in some patients 7.

### Recording in lab animals

According to the literature, recording VEMPs in lab animals is usually done invasively by means of leads placed directly on the target nerve, which is difficult because of the need for muscle contraction during the recording^36^, ^37^, ^38^, ^39^.

In a recent study^40^, the animals were previously trained, and this helped achieve a technique that would facilitate VEMP recording, directly on the muscle, without the need for invasive methods. In this study, it was easier to record VEMPs from the trapezius muscle thanks to the training method used with the animals in order to reach proper muscle contraction. In these recordings, the active leads were placed on the median posterior neck, 2cm apart from each other and equidistant from the central line of the spine; the reference one was placed on the medial portion of the chest and the ground electrode was placed on one of the animal’s foot or on the forehead. In order to train the animals we used a water reservoir, towards which we drew the animal’s nose, to force it to further extend its head, as a mechanism of self-protection against drowning, which caused the necessary muscle contraction for the test. Some animals adapted easily after some attempts and stood still with their heads out in order to keep their trapezius muscles stretched. However, in some cases, the animals were less cooperative, and even after many attempts they insisted on doing random head rotational movements. In these cases we replaced the lab animals by more docile beasts.

## DISCUSSION

In recent years, the study of vestibular evoked myogenic potentials has been greatly appreciated by scholars of neurotology from all over the world. In developed countries, especially in Europe and the USA, click-evoked myogenic potentials are been employed as a complementary test for neurotological investigations. However, in Brazil they are still rarely explored.

Despite being relatively old, discovered in the second half of the 50’s, this reflex is not well known, making up a universe of possible research and applications.

In some countries, the recording of these potentials have become common practice in the routine work of physicians and audiologists. Even if its structure is yet to be completely understood, given the little time since the scientific world has gotten an interest in this topic, the analysis of this reflex has brought about growing support to the diagnosis of severe pathologic conditions, such as tumors in the acoustic nerve (vestibular neuritis), Meniere’s disease, multiple sclerosis and sensorineural hearing loss^41^, ^42^, ^43^, ^44^, ^45^.

However, there is no standard method to carry out these tests, and numerous research projects are ongoing in an attempt to find the best method to record such potential.

In the literature^2^, ^8^, ^13^, ^14^, ^17^, ^19^, ^20^, ^25^, ^27^, ^28^, ^34^, it is reported that the trapezius and the sternocleidomastoid muscles allow a better recording of VEMPs, because it is easier to place the leads and there is higher muscle action potential triggering, necessary to obtain this potential, and its recording in the other anatomical regions is practically unfeasible.

As to stimuli averaging, tone burst stimuli need a lower triggering threshold than click evoking^46^, ^23^. The 500Hz tone-burst is clinically better because these VEMPs can be triggered by the lowest intensity possible stimuli^45^, ^46^, ^47^.

In most studies^2^, ^8^, ^9^, ^10^, ^11^, ^14^, ^15^, ^16^, ^18^, ^19^, ^24^, ^28^, ^29^, ^30^, ^31^, the stimuli were averaged by means of ear phones instead of the bone vibrators and the intensities were, usually, above 90 dBHL.

There is a wide range of choices regarding filters used to eliminate artifacts and recording windows for this potential; however, the ones most used are from 20 to 2000Hz^15^, ^16^, ^17^, ^18^, ^19^, ^22^, ^24^, ^27^, ^28^ and windows of 100ms^13^, ^14^, ^15^, respectively.

## CONCLUSIONS

There is no literature consensus as to the best method to record vestibular myogenic evoked potentials, and there is a need for more specific research in order to compare these recordings and to establish a standard to be used in the clinical practice.
